# Coupled Electronic and Ionic Conductivity in Strain‐Stiffening Hydrogels

**DOI:** 10.1002/advs.76247

**Published:** 2026-06-25

**Authors:** Md Al Raihan, Mark M. A. Mikhail, Khaled M. Hijazi, Hessameddin Yaghoobi, John P. Frampton, Vahid Adibnia, Michael S. Freund

**Affiliations:** ^1^ Department of Chemistry Dalhousie University Halifax Canada; ^2^ School of Biomedical Engineering Dalhousie University Halifax Canada; ^3^ Department of Biomaterials & Applied Oral Sciences Dalhousie University Halifax Canada; ^4^ Department of Biochemistry & Molecular Biology Dalhousie University Halifax Canada

**Keywords:** bioelectronics, biomaterials, boronic‐acid‐functionalized polyaniline, conjugated polymers, dynamic crosslinking, nanocomposite hydrogels, non‐linear rheology, polyvinyl alcohol

## Abstract

Advanced bioelectronics require soft materials that mechanically mimic tissues by exhibiting nonlinear mechanics and seamlessly bridge ionic signals in tissues and electronic signals in circuits. Creating conductive hydrogels with percolative electronic pathways that show tissue‐mimetic strain‐stiffening behavior represents a promising strategy to potentially address this need. Here, we report a composite hydrogel of poly(vinyl alcohol) (PVA) and poly(aniline boronic acid) (PABA) that exhibits strain‐stiffening mechanical behavior with mixed ionic and electronic conduction. Dynamic self‐healing boronic‐ester crosslinks that impart strain‐stiffening also facilitate the formation of a percolative network of conjugated conductive polymers during gelation, providing a continuous pathway for electronic transport (*σ_e_* ∼ 10^−5^–10^−3^ S m^−1^) alongside ionic conductivity (*σ_i_* ∼ 1–10 S m^−1^). As a result, deformation directly modulates the electronic resistance, displaying a distinct resistance‐stabilization plateau that coincides with the onset of strain‐stiffening, suggesting a transition from geometry‐limited to alignment‐assisted charge transport within the network. By unifying adaptive tissue‐like mechanics with dual conduction, this system offers a promising avenue for developing soft, mechanically resilient materials capable of continuous electromechanical transduction.

## Graphical abstract

A soft PVA–PABA hydrogel is developed that couples tissue‐like strain‐stiffening mechanics with dual ionic–electronic conduction. Dynamic boronic crosslinks enable self‐assembled percolative pathways, producing deformation‐responsive resistance with a stabilization plateau at stiffening onset, revealing alignment‐assisted charge transport and advancing resilient materials for continuous electromechanical transduction in bioelectronics.

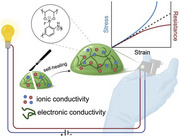

## 1 Introduction

Hydrogels, composed of hydrophilic networks of crosslinked polymers swollen in water, have attracted significant attention as soft, tunable platforms for biointerfaces, sensors, and soft robotics [1, 2, 3, 4]. Despite their desirable features—compliance, water retention, biocompatibility—there remains a key challenge in replicating the mechanical properties of biological tissues [5]. In nature, networks of fibrous macromolecules such as collagen, actin, and fibrin exhibit strain‐stiffening: a nonlinear increase in stiffness under increasing strain [6, 7]. This behavior enables tissues to remain pliable under low strain while offering protection against mechanical stress at higher strains through alignment and stretching of semiflexible filaments. The synthetic strain‐stiffening hydrogels that have been reported in the literature require precise molecular architectures to replicate the nonlinear mechanics of biopolymer networks [8, 9, 10, 11, 12, 13]. For example, polyisocyanopeptide gels exhibit thermally induced bundling of rigid helices [9], whereas bisurea‐based bolaamphiphile networks leverage confined bending modes in semi‐flexible micelles [8]. Dynamic covalent networks based on boronic ester formation have also emerged as a promising strategy, offering reversible, stimuli‐responsive crosslinking and the capacity for network reconfiguration under stress [14, 15]. Notably, strain‐stiffening polyethylene glycol (PEG) based boronic ester hydrogels were reported via reversible bond deformation and chain extension, eliminating the need for fibrillar structures [10]. Strain stiffening in both composite hydrogels and soft biological tissues follows universal scaling laws governed by matrix stretching and strain amplification [16]. These physical mechanisms provide a foundational framework for understanding nonlinear elasticity; however, the onset and magnitude of strain‐stiffening are highly sensitive to chemical composition, which determines the underlying network architecture, such as cross‐link density, chain length, and network homogeneity. Thus, identifying compositional variables that regulate dynamic crosslinking and network connectivity is central to designing synthetic hydrogels with tunable strain‐stiffening behavior.

Previously reported strain‐stiffening hydrogels are made of electronically insulating macromolecules [8, 9, 10, 11, 12, 13], limiting their utility in soft electronics and biointegrated devices where both mechanical adaptability and electrical conductivity are required. Strategies to impart conductivity into hydrogels have largely focused on the incorporation of conductive fillers (e.g., graphene, poly(3,4‐ethylenedioxythiophene):poly(styrenesulfonate) (PEDOT:PSS)) or ionic dopants (e.g., salts or acids) [17, 18, 19, 20, 21]. While effective in enhancing either electronic or ionic performance, these approaches generally introduce conductivity without directly controlling the nonlinear mechanical properties of the polymeric network. A recent study has introduced ion‐conducting strain‐stiffening hydrogels based on a composite architecture of poly(acrylic acid) and polyacrylamide, where mechanical nonlinearity arises from the embedded reinforcing domains in a patterned hydrogel [22]. However, ionic conductivity alone typically results in a slower charge transport and cannot efficiently couple to electronic devices [23], underscoring the need for complementary electronic pathways to enable faster, more efficient signal transduction [24, 25]. A similar need for coupled ionic and electronic transport also appears in membrane‐based artificial photosynthetic systems, which rely on hydrated polymer networks to support light‐driven redox reactions [26, 27].

Here, we report a synthetic hydrogel that integrates coupled ionic and electronic conductivity with dynamic strain‐stiffening. The network is composed of poly(vinyl alcohol) (PVA) and poly(aniline boronic acid) (PABA), where PABA plays a bi‐functional role: forming reversible boronic ester cross‐links with the diol groups of PVA and imparting electronic conductivity via its self‐doped conjugated backbone [28, 29]. In this system, PABA concentration and pH‐dependent boronate ester formation serve as compositional parameters that modulate crosslink density, network connectivity, and consequently the onset and extent of strain‐stiffening. These components together form the PVA–PABA hydrogel, an electronically and ionically conductive polymer network. The dynamic boronic ester linkages allow for network reconfiguration under deformation, contributing to the observed self‐healing and nonlinear mechanical response. Simultaneously, the π‐conjugated structure of PABA facilitates electron delocalization and percolation, while the hydrated self‐doped PVA–PABA matrix supports substantial ionic transport.

These features yield a hydrogel that combines adaptive strain‐stiffening with mixed ionic–electronic conduction. The resulting PVA–PABA hydrogel exhibits mechanical–electrical transduction, wherein mechanical deformation (e.g., finger or wrist bending) produces real‐time, measurable electrical signals. Notably, the coupling between nonlinear mechanics and charge transport leads to a self‐regulated electromechanical response, where resistance increases with strain in the linear regime but approaches a near‐plateau as the network enters the strain‐stiffening region. The interplay of dynamic covalent crosslinking, polymer alignment, and dual‐mode conduction constitutes a promising strategy for fabricating hydrogels with strain‐adaptive mechanical properties and signal stability, which are critical for applications in wearable sensing and bioelectronic interfaces [30, 31, 32].

## 2 Results and Discussion

### 2.1 Synthesis and Incorporation of PABA Into a PVA Network

**FIGURE 1:**
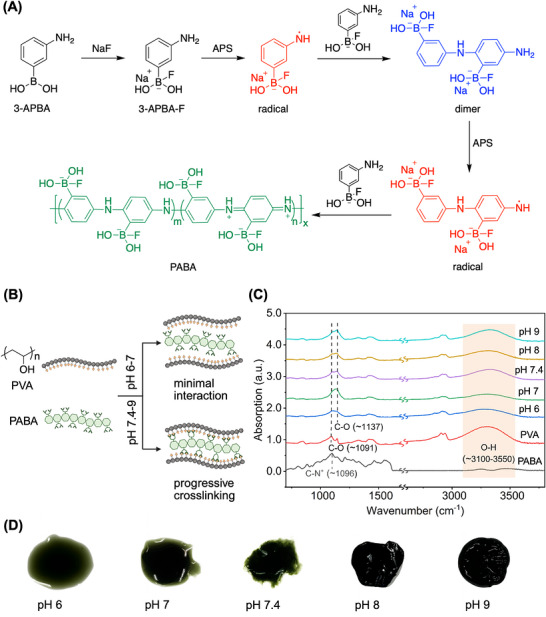
(A) Oxidative polymerization of 3‐aminophenylboronic acid (3‐APBA–F) yielding self‐doped PABA. (B) Schematic illustration of pH‐dependent boronate–diol crosslinking between PABA and PVA, showing minimal interaction at pH 6–7 and progressive crosslinking from pH 7.4 to 9 as the network develops. (C) ATR–FTIR spectra of PABA_0.30_ gels (pH 6–9) together with spectra of pure PABA and PVA. A shoulder at ∼1137 cm^−^^1^ emerges in the hydrogels, appearing adjacent to the PVA C─O stretching band at ∼1091 cm^−^^1^. This feature is attributed to C─O stretching associated with boronate ester linkages. (D) Optical images of PABA_0.30_ gels prepared at pH 6–9. The images were taken from hydrogel discs with a diameter of 2.0 cm.

PABA was synthesized via a modified oxidative polymerization of 3‐aminophenylboronic acid (3‐APBA) following reported methods [29, 33, 34]. Previous characterization of PABA synthesized using this fluoride‐assisted oxidative polymerization chemistry reported high molecular weight by high‐temperature GPC in NMP, with Mn ≈ 1.68 × 10^6^ g mol^−^^1^, Mw ≈ 1.76 × 10^6^ g mol^−^^1^, and Đ ≈ 1.05 [34]. Ammonium persulfate and sodium fluoride act as oxidant and activator, respectively, where fluoride coordination stabilizes tetrahedral boronate intermediates and promotes oxidative coupling near neutral pH. Figure 1A illustrates the oxidative polymerization route and resulting self‐doped PABA structure, in which iminium cations are stabilized by boronate–fluoride adducts. PABA was subsequently blended with an aqueous PVA solution to yield homogeneous PVA–PABA mixtures.

Following synthesis, ultraviolet–visible (UV–vis) spectra of diluted PVA–PABA mixtures prepared from the gel samples (Figure S1) show a broad near‐infrared polaron band near 800 nm at pH 6, characteristic of the electronically conducting emeraldine‐salt form of PABA. The band shifts slightly to ∼760 nm and remains largely unchanged between pH 7–10, indicating that the polaronic absorption, and thus the self‐doped electronic structure, is preserved under near‐neutral conditions. At pH > 10, the near infrared band diminishes while absorption near 600 nm emerges, consistent with deprotonation and conversion to the nonconducting emeraldine‐base form (Figure S1). This behavior parallels other observed self‐doped PABA dispersions [35], suggesting retention of self‐doping within the PVA network. The self‐doped structure of PABA adopts a tetrahedral boronate geometry that facilitates dynamic cross‐linking with PVA hydroxyl groups. To examine the influence of PABA concentration on network formation, hydrogels were prepared with 2.9 wt.% PVA and 0.15–0.30 wt.% PABA, with water comprising the balance. Samples are hereafter denoted as PABA_0.15_, PABA_0.20_, PABA_0.25_, and PABA_0.30_, corresponding to gels containing 0.15, 0.20, 0.25, and 0.30 wt.% PABA, respectively, with PVA concentration held constant at 2.9 wt.% in all samples.

The pH‐dependent gelation mechanism (Figure 1B) reflects a transition from weak boronate–diol associations at low pH to enhanced boronate ester crosslinking under alkaline conditions. While the schematic provides a simplified visual model, the underlying boronic ester chemistry between PABA and PVA is inherently dynamic and structurally heterogeneous. In the relaxed state, boronic acid sites on PABA can form cyclic boronate ester linkages with local 1,3‐diol units on PVA chains. Network formation arises from the multivalent nature of PABA, where multiple boronic acid units distributed along the polymer backbone can independently form reversible ester linkages with PVA diol groups. These reversible PVA–PABA boronate ester linkages allow the PABA backbone to function as a multivalent crosslinking motif, in which individual boronic acid sites dynamically exchange among intermolecular boronic ester crosslinks, local intramolecular loop structures, and non‐esterified boronic acid states. This reversible bonding equilibrium produces a structurally heterogeneous, pH‐responsive hydrogel network rather than a uniform static crosslinked architecture.

Attenuated Total Reflectance Fourier Transform Infrared (ATR–FTIR) spectra of the representative PABA_0.30_ gel across a pH range of 6–9 reveal the emergence of a shoulder at 1137 cm^−1^, which is not a prominent feature in either pure PVA or PABA and increases in intensity with increasing pH (Figure 1C). This feature appears as a shoulder to the PVA C–O stretching mode at 1091 cm^−1^ [36, 37], and is attributed to C─O stretching in boronate ester linkages, consistent with previous reports [38]. The progressive increase in intensity of this band with pH is consistent with enhanced boronate ester formation under more basic conditions. The rise of this feature parallels the macroscopic transition from weakly associated materials to more cohesive, self‐supporting gels observed in Figure 1D. The concordance between the spectroscopic evidence (Figure 1C) and the observed physical states of the gel (Figure 1D) supports the formation of a dynamically crosslinked network through boronic‐ester interactions.

### 2.2 Mechanical Analysis

Rheological properties of the hydrogels were analyzed for PABA_0.15_–PABA_0.30_. As shown in Figure S2, all samples exhibited elastic‐dominant behavior (*G*′ > *G*′′, where *G′* and *G′′* denote the storage and loss moduli, respectively), confirming the formation of crosslinked networks across the tested pH range of 6–9. The *G′* increased systematically with both PABA concentration and pH, consistent with the formation of more boronic ester crosslinks [39]. While lower PABA concentrations (e.g., PABA_0.15_–PABA_0.25_) produced soft gels (*G*′ ≈ 2–12 Pa) with a modest pH response, a significant mechanical enhancement was observed at the higher PABA concentrations. The PABA_0.30_ sample, for instance, exhibited a substantial increase in stiffness, with *G′* increasing from ∼12 Pa at pH 6 to ∼100 Pa at pH 9.

**FIGURE 2:**
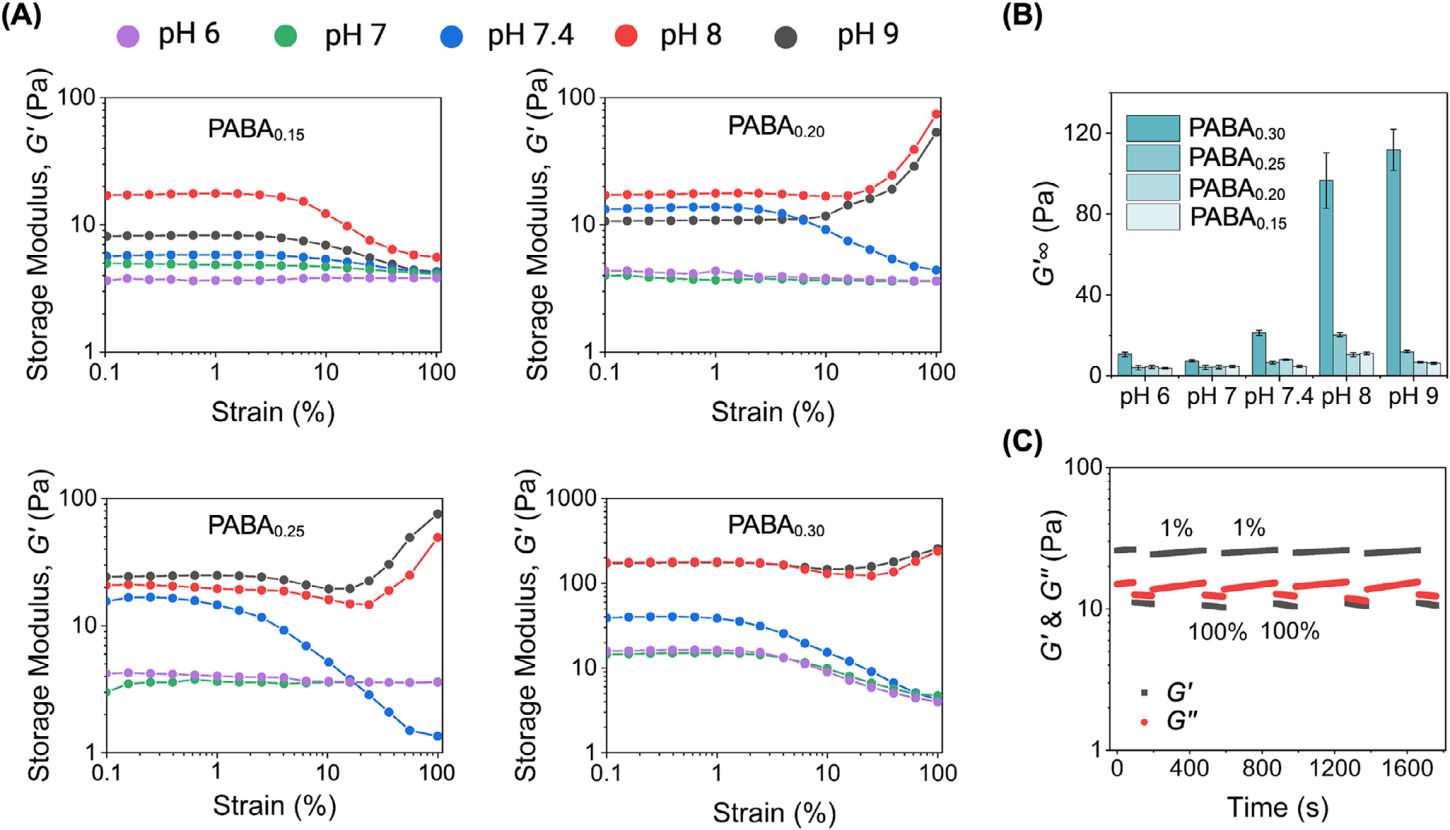
Viscoelastic properties of PVA–PABA hydrogels as a function of PABA concentration and pH. (A) Oscillatory strain sweeps (0.1%–100%) show *G′* for PABA_0.15_–PABA_0.30_ gels prepared at pH 6–9. (B) $G_{\infty }^{\prime }$ extracted from the linear regime for samples with different PABA concentrations, highlighting the sharp increase in the gel stiffness at pH 7.4–9. Error bars represent standard deviations from three independent measurements on three different samples. (C) Step‐strain recovery test of a PABA_0.30_ hydrogel at pH 8, alternating between large (100%) and small (1%) deformations at a frequency of 10 rad s^−1^, demonstrates reproducible restoration of *G*′ and *G*″, indicative of dynamic, self‐healing crosslinks.

The strain‐dependent mechanical properties of the hydrogels with different PABA concentrations at a pH range of 6–9 were studied by amplitude sweep mechanical tests (Figure 2A). At the lowest PABA content (PABA_0.15_), the small *G′* value of ∼10 Pa decreased rapidly beyond ∼10% strain, consistent with the hypothesis that the transient boronate ester bonds fail to sustain a continuous load‐bearing network. Increasing the concentration to PABA_0.20_ resulted in a more elastic network that was pH‐dependent. In the acidic and near‐neutral pH of 6–7.4, the network remained weak and showed shear‐thinning behavior beyond 10% strain, whereas at alkaline pH values of 8–9, networks with strain‐stiffening mechanical properties emerged [40]. At PABA_0.25_ and PABA_0.30_, the strain‐dependent mechanical properties were similar to those of PABA_0.20_ across pH 6–9 but with significantly higher storage moduli, consistent with the formation of a denser percolated network at higher PABA concentrations. The steady‐state storage modulus values in the linear viscoelastic regime (strain of 1 %), plateau moduli $\left(G_{\infty }^{\prime }\right)$, for all gel formulations at different pH values are summarized in Figure 2B. These results show that PABA concentration and pH act synergistically to govern mechanical properties. Sufficient crosslinking is required to achieve a more elastic network, while near‐alkaline conditions optimize boronate ester formation, yielding hydrogels that exhibit strain‐stiffening.

The nonlinear mechanical response under large deformation can also be seen in stress–strain curves (*σ*–*γ*) extracted from oscillatory strain sweep measurements for representative hydrogels (Figure S3A). The resulting curves exhibit a progressive increase in slope with increasing strain, indicative of nonlinear mechanics.

The viscoelastic behaviors of the crosslinked networks were further analyzed in frequency sweep tests for all hydrogel formulations (Figure S3B). The *G′* exceeded *G*″ across all studied frequencies (0.1 – 100 rad s^−1^), pH (6‐9), and PABA concentrations, confirming elastic‐dominant behavior. However, the frequency dependent *G*″ and *G*′ indicate that at pH values of 6 and 7, as well as for PABA_0.15_ and PABA_0.20_, weak networks of polymers are formed that are close to the percolation threshold of the crosslinked polymers, as indicated by the scaling relation $G^{\prime }∼G^{\prime \prime }∼\omega^{\alpha }$ observed at high frequency limits, where ω is angular frequency and *α* = 1.8 is the critical relaxation exponent [41, 42]. In contrast, PABA_0.25_ and PABA_0.30_ showed relatively less frequency‐dependent elastic behavior (less than 20% variation across the frequency range of 0.1 – 100 rad s^−1^) as expected for highly crosslinked networks of polymers.

These observations parallel those reported by Webber et al., who demonstrated strain‐stiffening in PEG–boronic ester gels through stress‐induced reconfiguration of dynamic covalent crosslinks [10]. In their system, gels formed over a broad pH range but exhibited weaker mechanical properties at mildly acidic conditions (pH < 7), consistent with reduced formation of tetrahedral boronate esters. A more pronounced stiffening response was observed at pH ≥ 7.4, where crosslinking via boronic ester exchange is favored. The pH‐dependent mechanical behavior observed in the PVA–PABA hydrogels similarly reflects the dynamic nature of boronic ester bonding, with strain‐stiffening emerging most clearly at pH 8 and 9.

Boronic ester bonding is reversible, allowing the formation of a network that dissipates energy by breaking the bonds between polymers under mechanical stress and reforming them at rest. The self‐healing behavior of the hydrogel was characterized for PABA_0.30_ at pH 8 using a step‐strain rheology test that alternates the applied strain between 1 and 100% at a frequency of 10 rad s^−1^ (Figure 2C). Under large strains of 100%, the *G′* decreased significantly to a value lower than the *G″*, reflecting disruption of crosslinking interactions. Upon application of a small‐amplitude shear with strain of 1%, both *G′* and *G″* rapidly recovered to near‐initial values, with full restoration achieved within a single cycle [43]. This recovery was repeatable over multiple cycles without appreciable fatigue, confirming that network integrity is maintained through reversible boronate ester exchange. In addition to rheological recovery, macroscopic cut‐and‐connect experiments further confirmed the self‐healing ability of the hydrogel, where two separated pieces rejoined after contact to form a continuous body (Figure S3C). These results highlight the ability of PABA_0.30_ hydrogel to repeatedly self‐heal under large deformations, consistent with the dynamic nature of its crosslinking chemistry [44].

**FIGURE 3:**
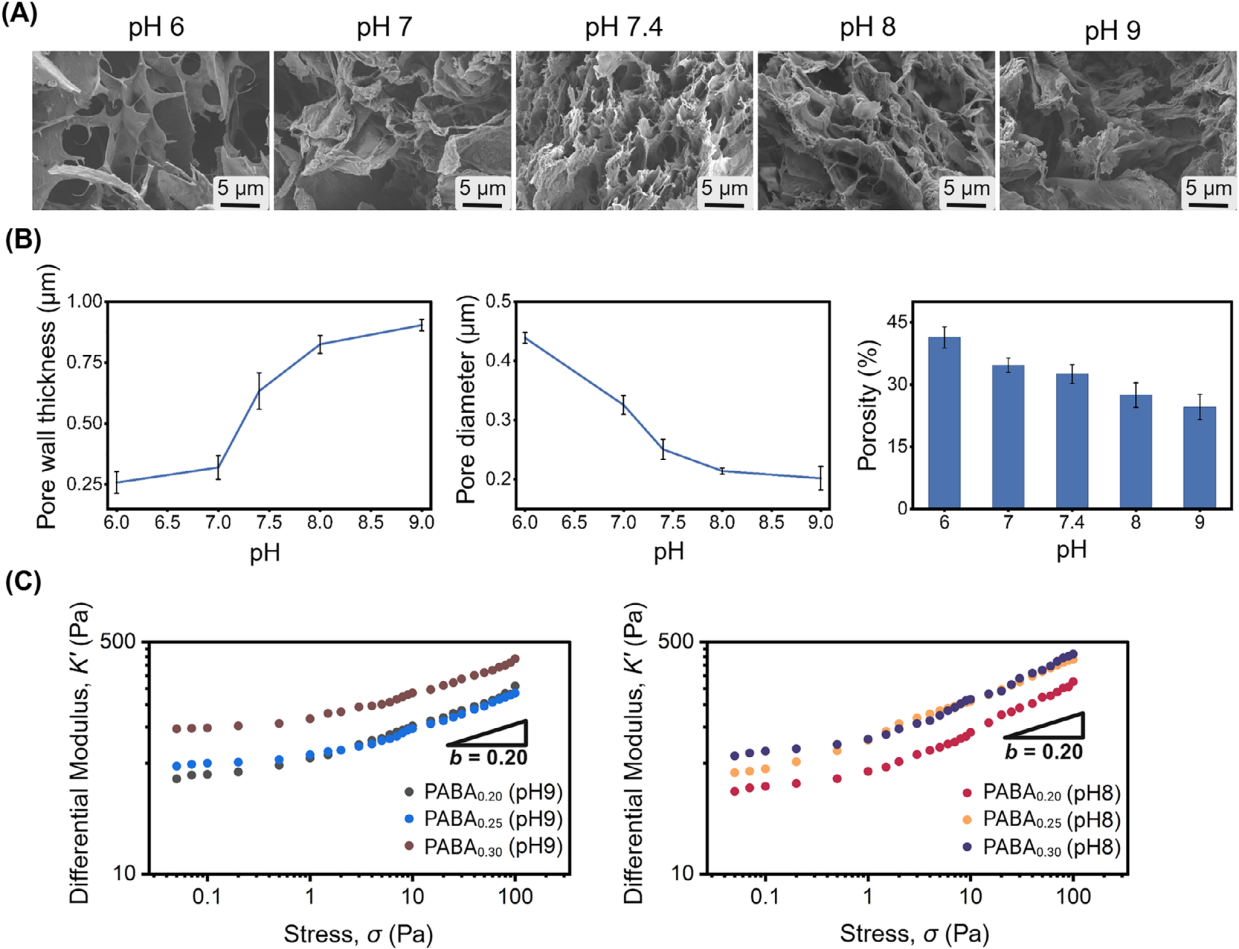
Microstructural and nonlinear mechanical properties of PVA–PABA hydrogels. (A) Representative SEM images of freeze‐dried PABA_0.30_ prepared at pH 6–9, showing a transition from an open network under acidic conditions to a dense network. Scale bars: 5 µm. (B) Quantitative analysis of pore diameter, pore wall thickness, and porosity as a function of pH (n = 3 independent samples; values are mean ± SEM, standard error of the mean). Average pore sizes decreased from 0.44 ± 0.01 µm at pH 6 to 0.20 ± 0.02 µm at pH 9, while pore wall thickness increased from 0.26 ± 0.04 µm to 0.90 ± 0.02 µm. Porosity decreases progressively from 41.34 ± 2.5% at pH 6 to 24.6 ± 3.1% at pH 9, supporting progressive network densification at higher pH. (C) Differential modulus (*K*′ = *dσ*/*dγ*) as a function of applied stress for PABA_0.20_–PABA_0.30_ hydrogels at pH 8 and 9. All formulations exhibit strain‐stiffening, with higher concentrations and alkaline conditions producing larger moduli.

The strain‐stiffening that was observed in Figure 2A can be further explained based on scanning electron microscope (SEM) images of the hydrogel microstructure. These images revealed a pronounced effect of pH on the hydrogel network microstructure (Figure 3A), which is quantitatively characterized in terms of pore diameter, pore wall thickness, and porosity in Figure 3B. Under acidic conditions (pH 6–7), the network exhibited large, irregular pores with relatively thin and loosely connected structures, consistent with a weakly crosslinked architecture. By contrast, gels formed under alkaline conditions (pH 8–9) displayed a more compact and highly interconnected network, characterized by reduced pore size and increased pore wall thickness. Although some features appear more fibrillar at higher pH, these correspond to interconnected pore walls rather than discrete fibers, reflecting enhanced network connectivity and densification. Quantitative analysis confirmed these observations. Pore diameters decreased by ∼50% from 0.44 ± 0.01 µm at pH 6 to 0.20 ± 0.02 µm at pH 9, while pore wall thickness increased from 0.26 ± 0.04 µm to 0.90 ± 0.02 µm over the same range. In parallel, porosity decreased progressively with increasing pH (Figure 3B, Table S1). The reduction in porosity is gradual between pH 6.0 and 7.4, followed by a more pronounced decrease under alkaline conditions, where porosity decreases to 27.44 ± 2.95% at pH 8.0 and 24.55 ± 3.06% at pH 9.0. This corresponds to an additional reduction of ∼16% and ∼25%, respectively, relative to pH 7.4, supporting the formation of a more densely crosslinked and compact network at higher pH.

These morphological transitions are consistent with nonlinear rheology. The nonlinear mechanical behavior of the PVA–PABA hydrogels was evaluated by measuring the differential modulus (*K*′ = *dσ*/*dγ*) as a function of shear stress (*σ*) (Figure 3C). The analysis revealed a power‐law scaling, *K*′ ∝ *σ^b^*, where *b* is the stiffening exponent (Table S2). All samples fitted in Table S2 showed *b* > 0, confirming nonlinear reinforcement. At pH 8, *b* values were ∼0.24–0.26, reflecting a moderate stiffening response, whereas at pH 9 the values decreased modestly to ∼0.16–0.20. Accordingly, b was extracted only for pH 8 and 9 samples, where clear nonlinear stiffening regimes were observed. This modest decrease is consistent with the observed microstructural changes. Denser, more crosslinked networks at higher pH exhibit higher baseline stiffness but may show a smaller relative increase in stiffness under applied stress, while looser, less crosslinked networks retain greater deformability and thus may stiffen more relative to the baseline under stress. This inverse relationship between initial stiffness and strain‐stiffening intensity parallels PEG–boronate ester hydrogels, where the stiffening exponent (*b* ≈ 0.17–0.49) decreases with increasing crosslink density [10]. The PVA–PABA gels exhibit similar *b* values compared to PEG–boronate ester hydrogels, suggesting that the underlying mechanism is similarly crosslink‐driven rather than entanglement‐driven. Noticeably, the exponents for both these synthetic boronate systems remain substantially lower than those of biopolymer networks such as actin, collagen, and fibrin (*b* ≈ 0.5–1.5) [44, 45], which stiffen primarily through the entropic stretching of aligned filaments. This mechanistic distinction also extends to other systems reliant on fibril alignment, including agarose (*b* ≈ 0.8), polyisocyanopeptides (*b* ≈ 1.5), and self‐assembled peptide or fiber gels (*b* ≈ 1.0) [8, 9, 46].

A further distinction lies in crosslinker architecture. PEG–boronate ester networks employ discrete, monomeric junctions (4‐arm PEG macromers), whereas the PVA–PABA system features a polymeric crosslinker in the form of PABA. Each PABA chain can engage in multiple, spatially distributed interactions with PVA, creating a dynamically connected network with distributed reversible crosslinking sites. This multivalent, polymeric crosslinker topology may facilitate load redistribution under strain. Mechanistically, applied stress induces alignment and stretching of PVA–PABA chains, producing both (i) entropic elasticity, from the reduction in chain conformational entropy, and (ii) enthalpic elasticity, from bond stretching within the polymer backbone and boronic ester linkages. The dynamic nature of boronic ester bonding allows the network to reorganize through reversible boronate exchange, progressively redistributing load among dynamic crosslinks [10]. SEM observations support this mechanism, showing a progressively denser and more interconnected network at higher pH, consistent with more extensive crosslinking and possible stress redistribution through a more highly crosslinked architecture. The slightly reduced *b* at high pH thus reflects a transition from a more deformable network to a pre‐reinforced and mechanically robust structure. The nonlinear mechanics of PVA–PABA hydrogels arise from reversible boronate reconfiguration within a pH‐tunable crosslinked network, providing adaptive elasticity distinct from fibrillar hydrogel systems.

The pH‐responsive network structure of the PVA–PABA hydrogels is expected to directly influence their electrical behavior. The self‐doped boronic acid groups along the PABA backbone not only act as dynamic crosslinking sites but also introduce intrinsic charge carriers capable of electronic conduction. At the same time, the ionic pathway is sustained by the transport of mobile ions (e.g., Na^+^, F^–^) through the hydrated PVA matrix, which maintains continuous ion conduction across the network. The coexistence of these complementary transport modes, electronic through the self‐doped PABA domains and ionic through the water‐swollen PVA network, results in a coupled mechano‐electronic response, which was further examined through its conductive properties.

### 2.3 Conductivity of PVA–PABA Hydrogels

**FIGURE 4:**
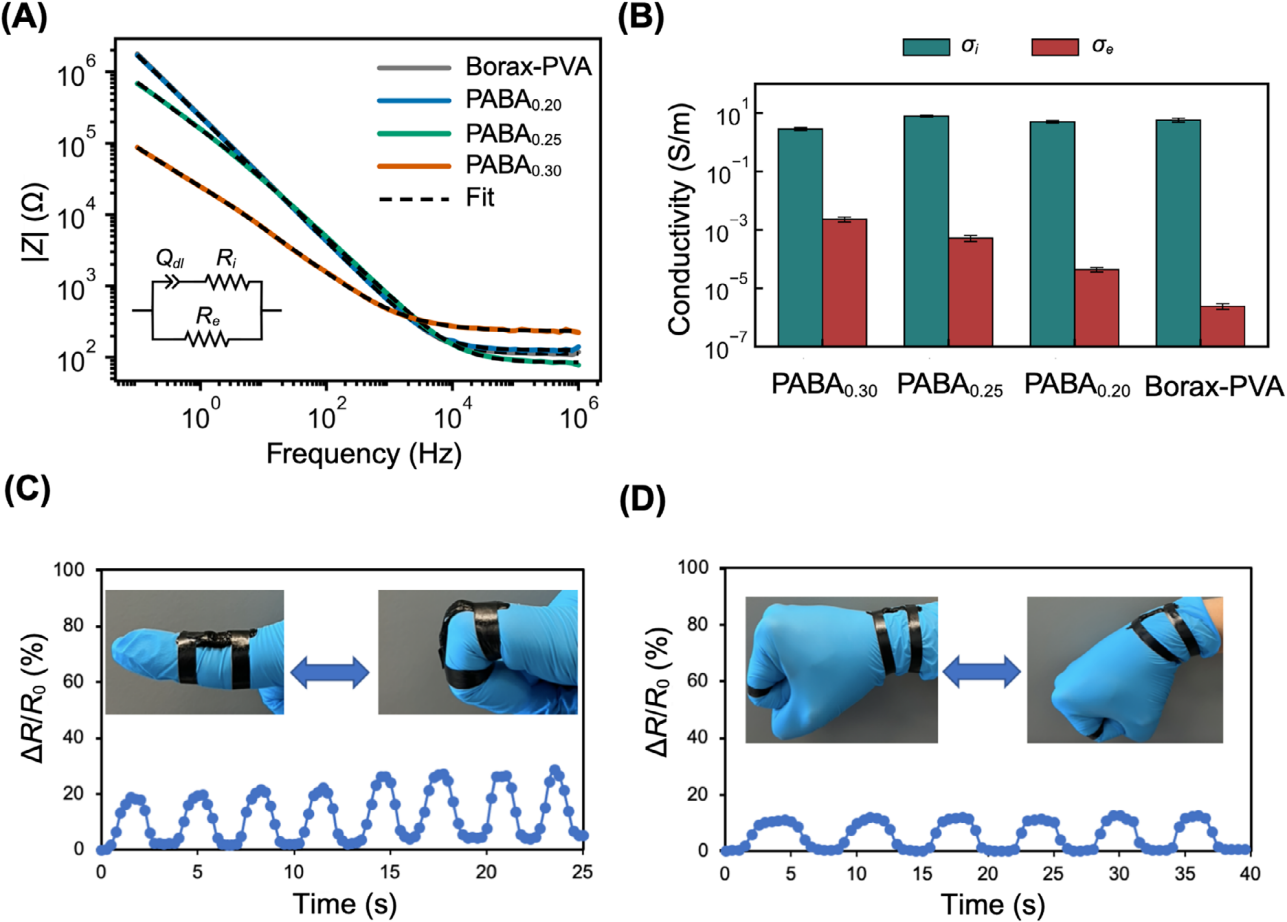
Electronic conductivity and strain‐sensing performance of PVA–PABA hydrogels at pH 8. (A) Bode magnitude spectra (solid) with RQ/R fits (dashed) used to decouple *σ_i_* and *σ_e_* contributions. (B) Extracted conductivities showing relatively stable *σ_i_* (1–10 S m^−1^) and composition‐dependent *σ_e_* (10^−5^–10^−3^ S m^−1^); control PVA–borax (pH 8) exhibited exclusively ionic transport. (C) Relative resistance change (Δ*R*/*R*_0_) of PABA_0.30_ hydrogel (pH 8) under cyclic finger bending. (D) Δ*R*/*R*_0_ response of the same hydrogel under cyclic wrist bending.

Electrochemical impedance spectroscopy (EIS) with an RQ/R equivalent‐circuit model was used to decouple ionic (*σ_i_*) and electronic (*σ_e_*) contributions to the total conductivity [47]. In this model, *R_i_* represents the ionic resistance, *R_e_* the electronic resistance, and *Q* the constant‐phase element describing interfacial dispersion. The overall impedance response and fidelity of the equivalent‐circuit fits are illustrated in the Bode magnitude spectra (Figure 4A), and the extracted conductivity values are summarized in the bar chart (Figure 4B). Representative Nyquist plots and corresponding fits are provided in Figure S4.

A control hydrogel with no electronic conductivity, PVA–borax, consisted of tetraborate decahydrate (borax, 0.5 wt.%) and PVA (4.0 wt.%) at a pH of 8. The hydrogel exhibits purely ionic transport mainly from Na^+^ ions, with minor contributions from borate species, yielding *σ_i_* of 5.62 S m^−1^ and *σ_e_* values (∼10^−6^ S m^−1^) (Table S3). These residual *σ_e_* values are reported solely for consistency in circuit fitting and are not interpreted as evidence of meaningful electronic transport, consistent with prior analyses of PVA‐based systems [47]. As an electronic‐material control, a dry PABA film showed a four‐point‐probe conductivity of approximately 330 S m^−^^1^, confirming that PABA is intrinsically electronically conductive, although this value is not directly comparable to the hydrated composite hydrogel measurements. Consistent with this intrinsic electronic conductivity, incorporation of PABA into PVA resulted in a clear, composition‐dependent enhancement in electronic conductivity for PABA_0.20_–PABA_0.30_ at pH 8, with *σ_e_* spanning 10^−5^–10^−3^ S m^−1^, while *σ_i_* of 1–10 S m^−1^ was comparable to that for the control ionic gel. This emergence of electronic transport coincides with a macroscopic structural transition at pH 8: samples with PABA concentrations less than 0.20 wt.% were in solution state, whereas those at and above PABA_0.20_ formed stable gels, with phase separation and sedimentation occurring above PABA_0.30_ (see Figure S5). This correlation suggests that the percolation threshold for both the PABA electronic network and the crosslinked polymer network lies near a PABA concentration of 0.20 wt.%. The invariance of *σ_i_* with PABA loadings reflects the similar ionic content under alkaline conditions for all samples, whereas the progressive increase in *σ_e_* with PABA concentration arises from the interconnection of electronically conductive percolated PABA domains that enable electronic transport [48].

To evaluate the mechano‐electronic sensing capability of the PABA_0.30_ hydrogel prepared at pH 8, real‐time resistance measurements were conducted during finger and wrist movements. Hydrogels were cast into thin films (3.5 mm thickness) and tested immediately after preparation. The hydrogel was mounted across the target joint and secured using conductive carbon tape to maintain stable electrical contact during deformation (Figure 4C,D). At room temperature, finger bending produced distinct resistance modulations over successive bending–release cycles, with ΔR/R_0_ values increasing from approximately 20% in the initial cycles to nearly 30% in later cycles (Figure 4C). Wrist bending also generated reproducible resistance changes, although with a smaller amplitude of approximately 12%–14% (Figure 4D), consistent with the lower effective strain imposed by wrist motion compared with direct finger bending. In both cases, the resistance returned close to the initial baseline after each release, and the baseline remained steady with minimal drift over the measurement period. The modest cycle‐to‐cycle variation in peak height is attributed primarily to unavoidable differences in manually applied bending angle and local strain distribution during each motion cycle.

Environmental adaptability was further assessed by performing repeated bending tests at 10 and 40°C (Figure S6) and comparing these results with the room‐temperature response shown in Figure 4C,D. For finger bending, the peak Δ*R*/*R*_0_ response at 40°C remained comparable to the room‐temperature response, reaching approximately 20%–28%, whereas the response at 10°C decreased to approximately 10%–18%. For wrist bending, the response was similarly maintained at 40°C, with peak Δ*R*/*R*_0_ values of approximately 12%–15%, compared with approximately 12%–14% at room temperature; at 10°C, the response decreased slightly to approximately 8%–11%. These results show that the hydrogel retains stable electromechanical sensing behavior at both elevated and reduced temperatures, although the lower response at 10°C may reflect reduced polymer‐chain mobility and slower charge transport within the hydrated matrix.

Long‐term immersion stability was further evaluated by incubating PABA_0.30_ hydrogels prepared at pH 8 in pH 8 and pH 9 buffers for ∼168 h. The hydrogels remained macroscopically intact with no significant mass loss (Figure S6B,C), whereas the hydrogel lost structural integrity within ∼14 h in a pH 7 buffer. EIS analysis showed that both ionic and electronic conductivities were retained after ∼168 h immersion in pH 8 buffer (Table S4), indicating stable mixed‐conducting behavior under mildly alkaline hydrated conditions.

This system distinguishes itself from conventional hydrogel‐based sensors in several key aspects. Many reported strain sensors rely on ionic conduction, embedded nanofillers (e.g., CNTs, graphene), or conductive polymers like PEDOT:PSS—approaches that often introduce heterogeneity or compromise mechanical softness [35, 49]. In contrast, the PABA_0.30_ hydrogel achieves intrinsic electronic conductivity via a filler‐free polymeric network, enabling both strain sensitivity and mechanical uniformity without additional components. A quantitative comparison with representative strain‐stiffening hydrogels is provided in Table S5. The PVA–PABA hydrogel combines a strain‐stiffening index of b ≈ 0.16–0.26 with mixed ionic/electronic conductivity (*σ_i_* ∼ 1–10 S m^−1^; *σ_e_* ∼ 10^−5^–10^−3^ S m^−1^), distinguishing it from previously reported strain‐stiffening hydrogels that are generally electronically insulating or primarily ionically conductive. While these bending tests demonstrate a geometry‐dependent sensing behavior, a controlled tensile experiment is required to reveal the relationship between strain‐stiffening and charge transport.

### 2.4 Strain‐Stiffening–Driven Electromechanical Response

**FIGURE 5:**
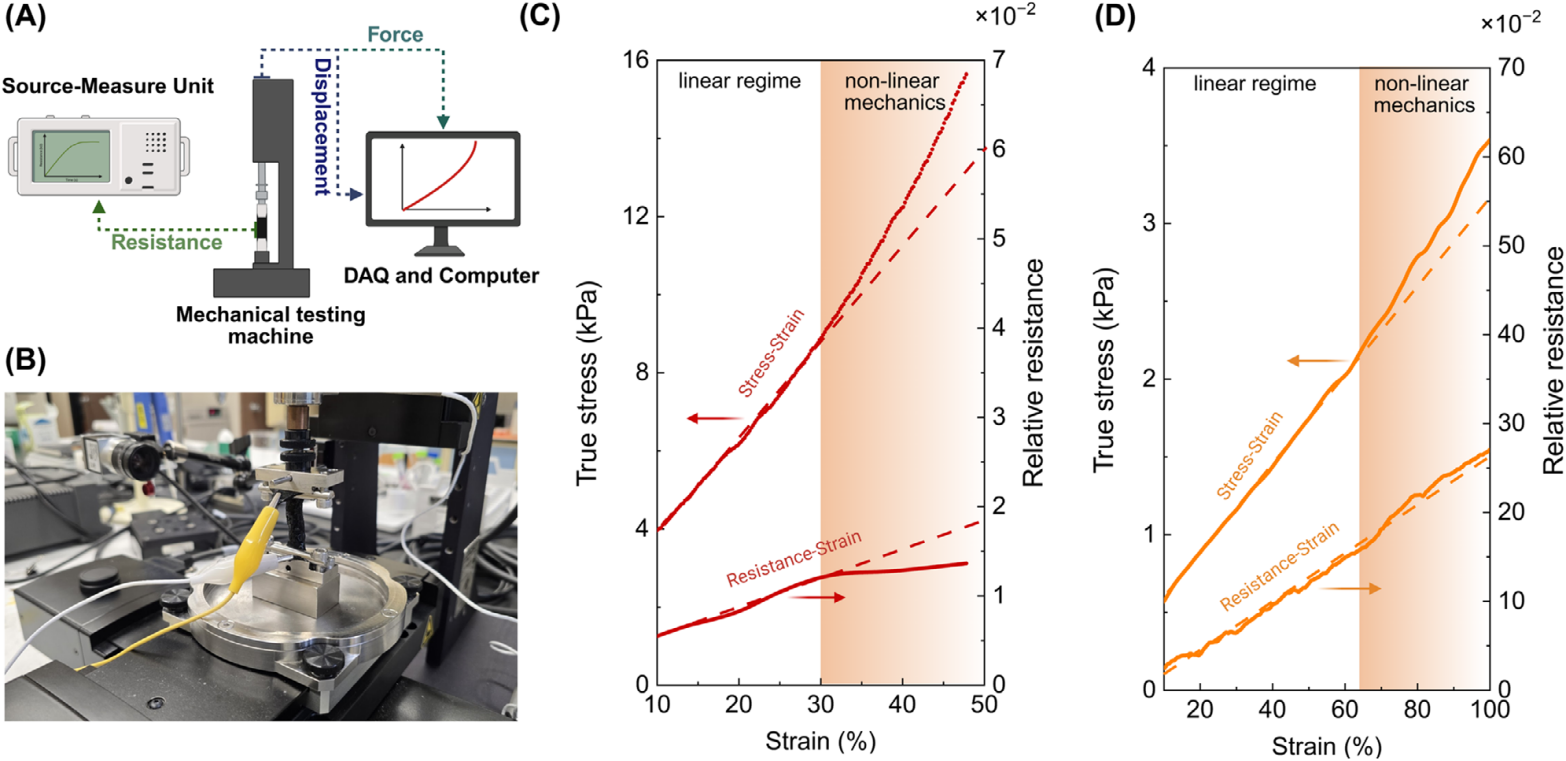
Strain‐dependent electromechanical response. (A) Schematic representation of the setup for synchronized true stress–strain and resistance measurements using a source‐meter coupled to a mechanical testing machine. (B) Photograph of the experimental configuration. (C) PABA_0.30_ hydrogel at pH 8 showing a resistance‐stabilization plateau in the strain‐stiffening regime. (D) PVA–borax (pH 8) control showing a continuous resistance rise consistent with purely ionic conduction. The dashed lines in C and D represent the best‐fit linear curves that represent the linear regime in both the stress‐strain and relative resistance‐strain curves.

The experimental setup for in situ electromechanical measurements is shown schematically in Figure 5A, with a photo of the setup shown in Figure 5B, wherein a mechanical testing apparatus was integrated with a digital source‐measure unit. This configuration enabled the simultaneous acquisition of stress–strain and relative resistance–strain data under uniaxial tensile deformation.

The PABA_0.30_ hydrogel exhibits the expected strain‐stiffening behavior above 30% strain, consistent with the rheology experiments (Figure 5C). A marked transition is, however, observed in the measured relative resistance as the hydrogel enters the nonlinear, strain‐stiffening regime. Within the linear viscoelastic region, the relative resistance increased linearly with the deformation. However, at the onset of strain‐stiffening, the rate of resistance growth decreased significantly, yielding a reproducible relative resistance plateau. This behavior was consistently observed across three independent samples (Figure S7). In contrast, the control PVA–borax (pH 8) hydrogel, which shows ionic conduction only, displayed a continuous linear increase in the relative resistance with strain (Figure 5D), irrespective of the viscoelastic regime.

We posit that at small deformations, the resistance increase is governed by geometric effects, elongation and narrowing of the conductive path, while the network elasticity is primarily entropic, arising from the conformational freedom of polymer chains. However, the simultaneous onset of the strain‐stiffening and the resistance plateau reflects a shared microstructural origin rooted in the transition from entropic to enthalpic elasticity within the dynamic boronate network. As the polymers and polymer bundles approach their extension (contour) limit, deformation becomes increasingly enthalpic, engaging bond stretching and promoting the directional alignment of the conjugated PABA segments. This microstructural evolution, which is consistent with the macroscopic strain‐stiffening response, may enhance interchain electronic coupling between PABA segments, thereby improving charge transport through interchain hopping pathways [50, 51]. The resulting improvement in electronic charge‐transport pathways may compensate, and at higher strains nearly offset, the continued geometric penalty. Such an entropic‐to‐enthalpic transition has been identified as the molecular basis of strain‐stiffening in boronate‐ester hydrogels [10]. However, the coupled mechanical and electrical responses observed here suggest a unique framework for mechano‐electrical signal transduction in highly stretchable electronically conductive materials.

This electromechanical behavior of the electronically conductive hydrogel is fundamentally different from that of an ionic strain‐stiffening hydrogel [52], as evident also in Figure 5D. In such ionic systems, stretching in the stiffening regime elongates ion transport pathways and reduces the cross‐sectional area, leading to a sharp increase in the relative resistance. Conversely, our findings share a conceptual parallel with PEDOT:PSS hydrogel [53] and stretchable PEDOT:PSS conducting polymer systems [54, 55], where strain‐induced conjugated chain alignment and crystallization enhance electronic charge transport, thereby mitigating the electrical effects of geometric thinning and corroborating the observed resistance‐stabilization plateau. Notably, this electromechanical behavior is achieved in a hydrogel that is resistant to cell adhesion (Figure S8A,B) and remains noncytotoxic over 7 days (see Section S1 for details) in culture with no evidence of harmful leachates (Figure S8C,D)—a combination that mitigates biofouling and suggests a low potential for irritation, making it suitable for external, skin‐interfacing bioelectronics.

**FIGURE 6:**
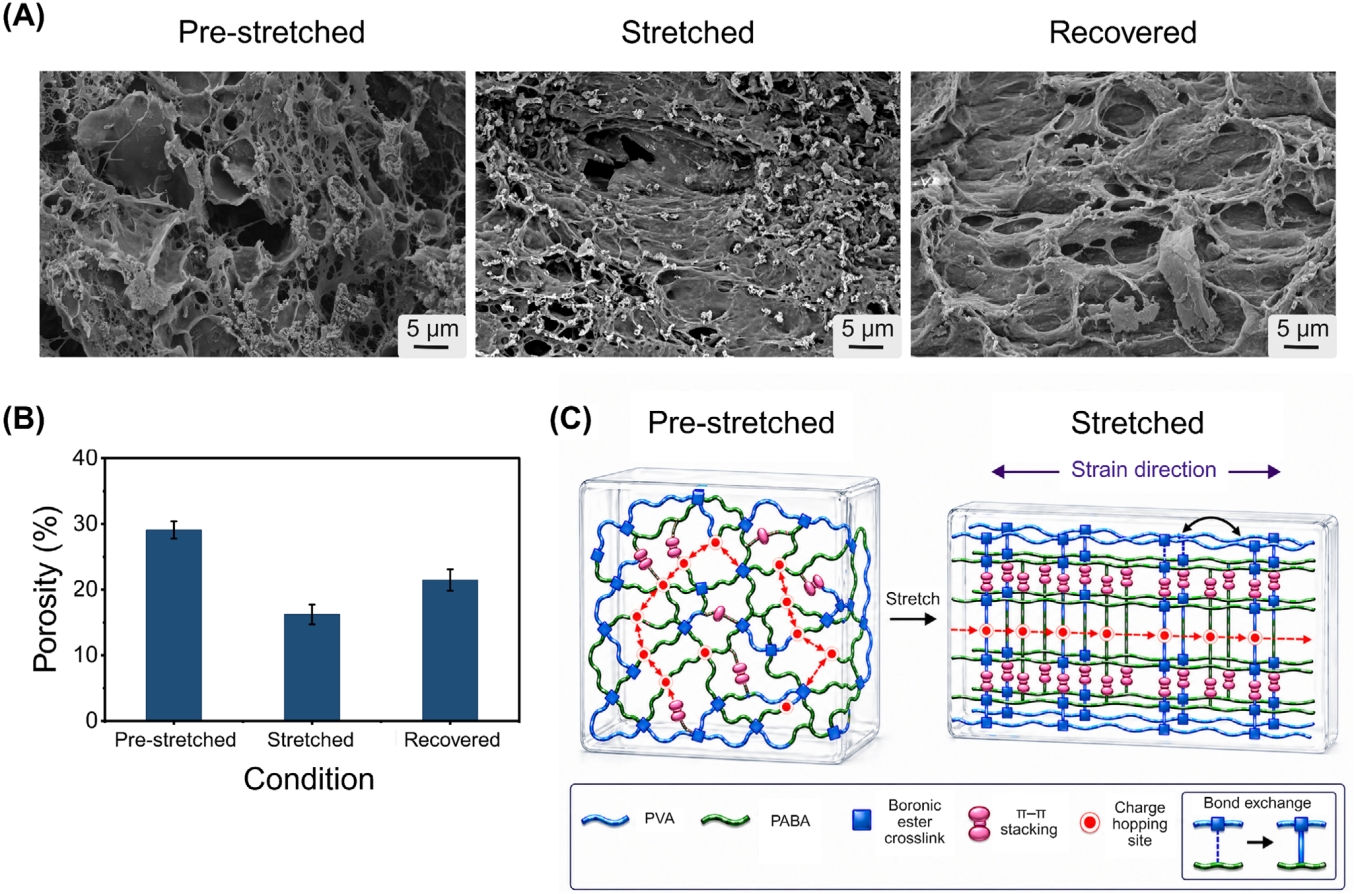
(A) SEM images of the PABA_0.30_ (pH 8) hydrogel in the pre‐stretched, stretched, and recovered states, showing pore collapse and alignment upon stretching and partial recovery after relaxation. Scale bars: 5 µm. (B) Porosity (%) derived from SEM images, showing a decrease upon stretching and partial recovery after relaxation (mean ± SEM, n = 3). (C) Schematic of strain‐induced microstructural reorganization, illustrating chain alignment, reduced interchain spacing, enhanced π–π interactions, and dynamic boronic ester bond exchange enabling reversible network rearrangement.

Structural insight into the resistance‐stabilization behavior observed in Figure 5C was sought by examining the microstructural evolution of the hydrogel under tensile deformation using SEM analysis and complementary schematic interpretation (Figure 6A–C). The deviation of the relative resistance response from the monotonic increase expected from purely geometric effects suggests that additional structural factors may influence charge transport under strain.

SEM analysis was performed on PABA_0.30_ (pH 8) hydrogels in the pre‐stretched, stretched, and recovered states (Figure 6A). The stretched state was prepared at approximately 40% tensile strain, within the nonlinear strain‐stiffening regime.

The initial hydrogel exhibits an open porous network, consistent with a hydrated, dynamically crosslinked structure (Figure 6A). Upon stretching, the porous architecture becomes compressed and undergoes preferential structural reorganization along the deformation direction, accompanied by partial pore collapse. After relaxation, the network partially recovers its porous morphology, although it does not fully return to the initial state, suggesting that deformation induces both reversible rearrangement and residual microstructural reorganization.

Quantitative porosity analysis supports these observations (Figure 6B). The porosity decreases from 29.1 ± 1.3% in the pre‐stretched to 16.3 ± 1.5% upon deformation, corresponding to an approximate 44% reduction, indicating significant densification of the network under applied strain. After relaxation, the porosity partially recovers to 21.5 ± 1.6% (≈74% recovery relative to the initial state), consistent with the dynamic, yet partially reversible nature of boronic ester crosslinks.

Based on these observations, we propose a strain‐induced microstructural reorganization mechanism that underlies the coupled electromechanical response (Figure 6C). As established by EIS analysis, charge transport in the initial (pre‐stretched) PABA_0.30_ hydrogel at pH 8 is dominated by ionic conduction through the hydrated network, while the measurable electronic conductivity confirms the presence of PABA‐based electronic transport pathways (Table S3). With stretching, polymer chains and interconnected pore walls undergo preferential structural reorganization along the strain direction, accompanied by microstructural densification and reduced apparent porosity. This structural rearrangement may reduce the effective spacing between PABA‐containing regions along the deformation axis and thereby influence interchain electronic coupling. Concurrently, dynamic boronic ester bonds undergo reversible exchange, enabling stress redistribution without catastrophic failure.

This microstructural evolution provides a plausible framework for the resistance stabilization observed during strain‐stiffening in Figure 5C. At low strain, resistance increases primarily due to geometric elongation and reduction of cross‐sectional area. In the nonlinear stiffening regime, strain‐induced alignment and densification may facilitate more favorable conduction pathways, partially compensating for the geometric resistance increase. The π–π interactions shown in Figure 6C are intended as a conceptual representation of possible electronic coupling between PABA segments, consistent with the view that strain‐stiffening and the associated stabilization of electrical response that arise from a combination of reduced porosity, dynamic boronic ester exchange, and deformation‐induced reorganization of conductive domains.

## 3 Conclusion

This study introduces a fully synthetic non‐cytotoxic hydrogel that intrinsically couples the strain‐stiffening capabilities with mixed ionic and electronic conductivity. The dynamic covalent crosslinking between PVA and PABA generates a dynamic crosslinked network that supports both mechanical and charge transport. The strain‐stiffening response (*b* ≈ 0.16–0.26) arises from reversible boronate ester reconfiguration within a pH‐dependent polymer network, while electronic conduction (*σ_e_* ∼10^−5^–10^−3^ S m^−1^) emerges at the gel regime, consistent with a shared percolation threshold for network mechanics and PABA connectivity. The unique strain‐insensitive resistance observed in the nonlinear viscoelastic regime for these gels suggests an intrinsic coupling between network mechanics and charge transport, where deformation‐induced alignment offsets geometric resistance effects. These findings provide insights for future design of adaptive, multifunctional hydrogels for applications such as wearable electronics, where soft, stretchable conductive substrates are of interest.

## Author Contributions

**Vahid Adibnia**: funding acquisition, conceptualization, writing – review and editing, resources, supervision, methodology, writing – original draft. **Mark M. A. Mikhail**: investigation, methodology, writing – review and editing. **John P. Frampton**: writing – review and editing, supervision, funding acquisition, methodology. **Khaled M. Hijazi**: methodology, writing – review and editing, data curation. **Hessameddin Yaghoobi**: methodology, writing – review and editing, data curation, visualization. **Michael S. Freund**: conceptualization, funding acquisition, writing – original draft, methodology, writing – review and editing, supervision, resources. **Md Al Raihan**: investigation, methodology, formal analysis, writing – original draft, writing – review and editing, data curation, visualization.

## Conflicts of Interest

The authors declare no conflicts of interest.

## Supporting information




**Supporting File**: advs76247‐sup‐0001‐SuppMat.docx.

## Data Availability

The data that support the findings of this study are available from the corresponding author upon reasonable request.
