# Supplementary material for: Coupled Electronic and Ionic Conductivity in Strain‐Stiffening Hydrogels
Source: Adv Sci (Weinh). 2026 Jun 25;13(52):e76247. doi: 10.1002/advs.76247 (PMC13336551; doi:10.1002/advs.76247)
Supplement: Supplementary file 1 [file ADVS-13-e76247-s001.docx]

**Supporting Information**

**Coupled Electronic and Ionic Conductivity in Strain-Stiffening Hydrogels**

Md Al Raihan,^1^ Mark M. A. Mikhail,^1^ Khaled M. Hijazi,^2,4^ Hessameddin Yaghoobi,^2^ John P. Frampton,^2,3^ Vahid Adibnia,^1,2,4*^ Michael S. Freund,^1*^

^1^Department of Chemistry, Dalhousie University, Halifax, B3H 4R2, Canada

^2^School of Biomedical Engineering, Dalhousie University, Halifax, Canada, B3H 4R2

^3^Department of Biochemistry & Molecular Biology, Dalhousie University, Halifax, Canada, B3H 4R2

^4^Department of Biomaterials & Applied Oral Sciences, Dalhousie University, Halifax, Canada, B3H 4R2

^*^Author to whom correspondence should be addressed.

**The Supporting Information includes:**

S1. Experimental Section

S2. Supplementary Figures S1 to S8

S3. Supplementary Tables S1 to S5

S4. References

**S1.** **Experimental Section**

**PVA-PABA Hydrogel Solution Preparation**

Polyvinyl alcohol (PVA, (Mw 85,000–98,000, 99+% hydrolyzed), Sigma-Aldrich) (0.97 g) was fully dissolved in 50 mL of Milli-Q water at 90 °C under continuous stirring. The solution was then allowed to cool to room temperature before further processing. Separately, 0.10 g of 3-aminophenyl boronic acid (3-APBA, Sigma-Aldrich), 0.19 g of sodium fluoride (NaF, Sigma-Aldrich), and 0.16 g of ammonium persulfate (APS, Sigma-Aldrich) were ground using a mortar and pestle for 15–20 minutes, during which the mixture gradually developed a blue-green color. The ground mixture was then dispersed in 10 mL of Milli-Q water and subjected to ultrasonication for 30 minutes, leading to a progressive color change from blue to green.

This 10 mL reaction mixture was slowly added dropwise to the cooled 50 mL PVA solution in a sonication bath. After complete addition, the mixture was stirred continuously overnight to form a green homogeneous solution. The following day, excess water was removed by controlled evaporation at 50 °C on a hot plate equipped with gentle magnetic stirring (300-400 rpm) for ~5-6 h until the final volume reached 32 mL. This procedure resulted in a final composition of 0.30 wt % PABA and 2.9 wt % PVA.

Similar to the preparation of 0.30 wt % PABA with 2.9 wt % PVA, additional formulations containing 0.25, 0.20, and 0.15 wt % PABA were prepared by maintaining the same procedure while adjusting the amounts of 3-APBA, NaF, and APS proportionally. The PVA concentration was maintained at 2.9 wt % across all samples. For clarity, samples are denoted as PABA_0.15_, PABA_0.20_, PABA_0.25_, and PABA_0.30_, corresponding to hydrogels containing 0.15–0.30 wt % PABA (with 2.9 wt % PVA and water balance).

**Hydrogel preparation process**

The as-prepared PVA–PABA precursor solutions exhibited an initial pH of approximately 5, consistent with acidification arising from proton release during oxidative polymerization and sulfate formation from persulfate reduction. To obtain hydrogels at defined pH values (6, 7, 7.4, 8, and 9), 0.1 M NaOH (Sigma-Aldrich) was added dropwise under continuous stirring until the desired pH was reached. For all PABA formulations (PABA_0.15_–PABA_0.30_), the pH was increased to promote boronic-ester cross-linking, thereby enabling gel formation under controlled conditions.

**Preparation of Borax–PVA Hydrogel**

A 4.5 wt % PVA solution (10 mL) was prepared by dissolving PVA in distilled water at 90 °C under continuous stirring until a clear solution was obtained. Separately, a 4.5 wt % sodium tetraborate decahydrate (Borax, The Dial Corp.) solution (10 mL) was prepared in water at 50 °C. The two solutions were mixed by syringe at a 1: 8 volume ratios (Borax: PVA), yielding a final composition of 0.5 wt % borax, 4.0 wt % PVA, and water as the balance.

**Rheological Characterization**

Oscillatory rheology was performed on a Discovery HR-2 rheometer (TA Instruments, USA) equipped with a 20 mm parallel plate geometry. To minimize solvent evaporation during measurements, the exposed sample edges were sealed with silicone oil following trimming. Amplitude sweeps were conducted at an angular frequency of 10 rad s^–1^ over a strain range of 0.1–100%. Frequency sweeps were performed at 1.0% strain over the range 0.1–100 rad s^–1^.

**Scanning Electron Microscopy (SEM) and Image Analysis**

Freeze-dried PVA–PABA (PABA_0.30_) hydrogels prepared at pH 6, 7, 7.4, 8, and 9 were sputter-coated with a standard ∼10 nm layer of gold prior to imaging. SEM micrographs were collected on a JEOL 840 (JEOL Ltd., Tokyo, Japan) operated at 5.00 kV using the SE2 detector (working distance 10.9 mm; magnification ∼2,000×). Pore diameters and wall thicknesses were quantified in ImageJ using the embedded 5 µm scale bar for spatial calibration. Measurements were performed manually by selecting a calibrated linear region within each image, from which ten representative pores and ten wall segments were measured consistently for each pH condition.

**Electrochemical Impedance Spectroscopy (EIS)**

EIS measurements were performed using a PARSTAT PMC-2000A potentiostat/galvanostat (Ametek, Princeton Applied Research, USA). Hydrogel samples were prepared immediately prior to testing, molded into cylindrical disks (1–4 mm thickness) using a Swagelok fitting, and the thickness was confirmed by calliper measurement before transfer to the measurement cell. For impedance measurements, the gels were placed into a separate Swagelok body equipped with two gold electrodes (CH Instruments, 2 mm diameter, electrode area = 0.031 cm²) inserted from opposite ends. Electrodes were gently advanced until a stable impedance response was obtained, ensuring full contact without additional compression. Impedance spectra were collected over the frequency range 0.1 Hz–1 MHz with a 10 mV AC perturbation at open-circuit potential.^[1]^ All measurements were conducted at room temperature (22–25 °C) on freshly prepared, fully hydrated samples to minimize drying artifacts.

**Tensile testing**

Tensile tests of PABA_0.30_ (pH 8) specimens (n=3) and Borax-PVA specimens (n=1) were conducted using Biomomentum Mach 1 universal testing system (Biomomentum, Canada). The setup can be seen in Figure 5A (main text). A 1.5 N single-axis load cell (MA999, Biomomentum, Laval, Canada) was installed to read the force (*F*) values. Specimens’ ends were gripped into the testing machine using pressure bar sample holders, leaving about 2 mm of the specimen ungripped (Figure 5B main text). After mounting them, pictures of the specimens were taken using a colour camera mounted next to the testing region, with a ruler placed adjacent to the specimens. These images were exported to Fiji, an open-source image analysis software,^[2]^ where the thickness of the specimens was measured at different cross-sections, followed by finding the average thickness (*T*) for each specimen. Given that the specimens had a semi-cylindrical shape, the average cross-sectional area (*A*) was assumed to be circular in shape and was calculated using the following equation:

$$A= \pi\left( \frac{T}{2} \right)^{2}$$

(1)

For each test, the distance between the two grips were measured using a Vernier calliper. Then the specimen was preloaded to 0.02 N to remove slack. The gauge length (*L*) was found by adding the displacement to the previously measured distance. Loading was then applied to all the samples until the force reached 0.4 N, the failure of the samples, or until the samples slipped out of the sample holders. The crosshead speed for all tests was 0.1 mm/s, with the data recorded at a frequency of 100 Hz. Time, in seconds, was recorded during the test.

After each test, the resistance and mechanical tests were analyzed. The force (*F*) – displacement ($\Delta L$) readings were converted to nominal stress ($\sigma_{nominal}$) and strain ($\varepsilon$) using the following equations:

$$\varepsilon= \frac{\Delta L}{L}$$

(2)

$$\sigma_{nominal} = \frac{F}{A}$$

(3)

The nominal stress assumes that the cross-sectional area of the specimens remained constant, which is not accurate. Hence, the true stress ($\sigma_{true}$) was calculated as described in previous work.^[3]^ The stretch ($\lambda$), which is described as the ratio between the instantaneous length and the initial length, is first calculated as follows:

$$\lambda= \frac{L + \Delta L}{L}$$

(4)

True stress considers the deformation of the specimen as stress increases and is described as follows:

$$\sigma_{true} = \lambda\cdot\sigma_{nominal}$$

(5)

The $\sigma_{true}$– time and $\varepsilon$– time were then filtered to match the lower frequency of the relative resistance-time, this was used to plot the $\sigma_{true}$ – $\varepsilon$ and the relative resistance – $\varepsilon$ (Figure 5C,D main text). The elastic modulus was calculated by measuring the slope of the $\sigma_{true}$ – $\varepsilon$ curves within the linear region. The trends of the relative resistance were observed with the deformation of the hydrogels.

**Electrical Measurements**

Resistance measurements were performed using a Keithley 2450 source-meter (Tektronix, Inc., USA) in a two-probe configuration, synchronized with the mechanical testing setup or finger/wrist motion to record real-time resistance changes during deformation. The relative resistance change (Δ*R*/*R*₀) was calculated as (*R* – *R*₀)/*R*₀, where *R* is the instantaneous resistance under strain and *R*₀ is the initial resistance at rest. For finger/wrist bending tests, hydrogel samples were positioned across finger or wrist joints, with both ends secured using conductive carbon tape to ensure stable electrical contact and connection to the source-meter.

**Preparation of PVA-PABA Films for Cell Culture**

The solution of PABA_0.30_ (pH 7) was spread onto circular glass coverslips using a pipette to form a layer of ~ 6 µm in thickness. Volumes of 150 µl and 60 µl were required for coverslips of 18 mm and 12 mm diameter, respectively. The samples were left at room temperature overnight to fully dry into films. The films with larger surface areas were used as produced while the films with smaller surface areas were taken off the coverslips and mounted on hanging cell culture inserts (0.4 μm pore size, VWR).

**Cell Culture and Staining**

Human dermal fibroblasts (WS1; CRL-1502, ATCC) were maintained in Eagle's Minimum Essential Medium (EMEM) with 1.5 g/L sodium bicarbonate, NEAA, L-glutamine, and sodium pyruvate (Corning™) supplemented with 1% antibiotic-antimycotic solution and 10% fetal bovine serum in a humidified incubator at 37˚C. WS1 cells were either cultured directly on the PABA_0.30_ films or on the surface of tissue culture plastic well plates in the presence of films contained in hanging cell culture inserts. Before starting the cell culture, PABA_0.30_ films mounted on coverslips and inserts were washed with 70% ethanol for 30 min. The ethanol was then removed and the films were washed five times with sterilized 1X Dulbecco's Phosphate-Buffered Saline (DPBS). In both cases, cells cultured on tissue culture plastic without PABA_0.30_ films or hanging cell culture inserts served as controls. WS1 cells were seeded at a density of 5×10^4^ cells per well of a 12-well plate.

After 3 and 7 days in culture, the cells were fixed with 4% paraformaldehyde (Sigma-Aldrich) in 1X DPBS for 15 min, washed with 1X DPBS, and then permeabilized using 0.25% Triton X-100 (Sigma-Aldrich) for 10 min. The cells were then washed with 1X DPBS and Atto 488-phalloidin (0.02 nM in PBS; Sigma-Aldrich) was added, followed by overnight incubation at 4°C. After incubation, the phalloidin stain was removed and the cells were washed with 1X DPBS. Hoechst 33342 (2 µM in PBS; Sigma-Aldrich) was then added for 15 min to stain the nuclei, followed by another wash with 1X DPBS. Images were obtained by epifluorescence microscopy using a Nikon Eclipse Ti microscope. From these images, the number of cells per unit area was quantified using the StarDist tool plug-in in Fiji.^[2]^

**Cytocompatibility of PVA-PABA Films**

WS1 fibroblasts grown on PABA_0.30_ films exhibited limited cell attachment (Figure S8A). This suggests limited adsorption of proteins present in the cell culture medium and may suggest applications where biofouling is a concern, e.g., implanted medical devices. However, the few cells that did attach to the films displayed morphologies similar to WS1 cells cultured on tissue culture plastic or glass (Figure S8B). The limited cell attachment may be due to the absence of integrin-binding sites necessary for cell adhesion, as the films were not treated with extracellular matrix (ECM) proteins such as collagen or fibronectin known to promote cell adhesion. For future application where cell attachment is desired, it may be possible to functionalize the PABA_0.30_ surface with ECM protein to promote cell adhesion/growth.

To gain a better understanding of the cytocompatibility of the samples, hanging cell culture inserts in which cells are not in direct contact with the material but share a fluid environment with the material were analyzed. As shown in Figures S8C and S8D, there was a significant increase in cell number from day 3 to day 7 for cells cultured on both tissue culture plastic alone (control) and in the presence of the PABA_0.30_ films. There were no significant differences between the number of cells attached to the tissue culture plastic in the presence of the PABA_0.30_ film and the control on day 3. However, WS1 cells proliferated slightly more for the control than the PABA_0.30_ films by day 7. These results indicate that PABA_0.30_ is not directly cytotoxic and likely does not release cytotoxic byproducts up to 7 days in culture. Future studies may explore how the material behaves in vitro with additional cell types and when placed subcutaneously.

**S2. Supplementary Figures**

**Figure S1.** UV–vis spectra of diluted PVA–PABA gels recorded at different pH values (6–14). A broad NIR polaron band centered near 800 nm characteristic of the emeraldine-salt forms up to pH 10, while the emergence of an absorption band near 600 nm above pH 10 indicates conversion to the emeraldine-base.

**Figure S2.** Time-dependent storage (*G′*) and loss (*G″*) moduli of PVA–PABA hydrogels measured at 21 °C under constant strain (1%) and angular frequency (10 rad s^–1^). Each hydrogel formulation contained 2.9 wt % PVA and increasing PABA loadings (PABA_0.15_–PABA_0.30_), as indicated above each column. The upper and lower rows correspond to *G′* and loss *G′′* moduli, respectively. Measurements were performed across five pH conditions (6–9). Elastic-dominant behavior (*G′* > *G″*) was observed in all cases, indicating network formation.

**Figure S3.** (A) Stress–strain relationships (*σ*–*γ*) derived from oscillatory strain sweep measurements for PVA–PABA hydrogels (PABA_0.20_–PABA_0.30_) at pH 8 and 9. The progressive increase in slope with strain indicates nonlinear mechanical reinforcement consistent with strain-stiffening. (B) Frequency-dependent *G′* and *G″* moduli of PVA–PABA hydrogels measured at 21 °C across formulation with PABA loadings (PABA_0.15_–PABA_0.30_), as indicated above each column and pH values (6.0–9.0). Measurements were conducted over an angular frequency range of 0.1–100 rad s^–1^ at 1% strain (within the linear viscoelastic regime). *G′* consistently exceeds *G″* across all conditions, confirming elastic-dominant behavior. (C) Visual demonstration of self-healing of the PABA_0.30_ (pH 8) hydrogel. The gel was cut, separated, and subsequently rejoined, showing recovery of structural integrity.

**Figure S4.** Nyquist plots of PVA–PABA hydrogels and control Borax–PVA (pH 8). Experimental data (solid lines) and RQ/R fits (dashed) for Borax–PVA, and PVA–PABA formulations (PABA_0.20_–PABA_0.30_, pH 8). The main plot shows the full impedance range, and the inset highlights the low-resistance region.

**Figure S5.** Photographs of vials containing 2.90 wt % PVA with increasing PABA loading (PABA_0.15_–PABA_0.35_). At PABA_0.15_, the sample remained a free-flowing solution, indicating that the percolation threshold had not been reached. Formulations PABA_0.20_–PABA_0.30_ formed self-supporting gels, whereas PABA_0.35_ exhibited sedimentation.

**Figure S6.** Environmental adaptability and immersion stability of the PABA_0.30_ hydrogel prepared at pH 8. (A) Relative resistance change (Δ*R*/*R*₀, %) under repeated mechanical deformation. Finger bending (left) and wrist bending (right) responses were recorded at 40 °C (gray) and 10 °C (blue). All measurements were performed over 20 bending cycles. (B) Optical images of the hydrogel immersed in pH 8 and pH 9 buffers over ~168 h, showing retention of macroscopic structural integrity at 0, ~24, ~72, ~120, and ~168 h. The hydrogel immersed in pH 7 buffer lost structural integrity within ~14 h, consistent with reduced boronate ester stability under neutral conditions. (C) Mass retention of the hydrogel immersed in pH 8 and pH 9 buffers over ~168 h, normalized to the initial mass at 0 h. Small fluctuations around 100% are attributed to hydration/swelling and handling during weighing.

**Figure S7.** Reproducibility of the stress–strain and resistance–strain responses of PABA_0.30_ hydrogel at pH 8. Data from three independent samples are shown in panels A–C. Solid lines represent experimental measurements, with true stress plotted against the left y-axis and relative resistance plotted against the right y-axis. Dashed lines represent linear fits to the initial linear regime for both the stress–strain and resistance–strain responses. Direct labels identify the mechanical stress–strain and electrical resistance–strain curves in each panel.

**Figure S8.** (A) Films placed on glass coverslips (left column) and hanging inserts (right column). (B) Limited cell attachment on the films on the coverslip at Day 3. (C) Number of WS1 fibroblast cells per unit area at day 3 and day 7 (control is tissue culture plastic). (D) Representative images of (C) which shows cells grow in the media in presence of the films. Values are expressed as Average ± SD (at least 7 spots on 3 wells for cell counting); *p < 0.05, ns: non-significance. Scale bar in B and D corresponds to 100 µm for all the images.

**S3. Supplementary Tables**

**Table S1.** Pore wall thickness, pore size, and porosity of PABA_0.30_ hydrogels across pH 6–9 (ImageJ Analysis of SEM Micrographs). Three independent samples (n = 3) were analyzed per pH, with ten ImageJ measurements per replicate. Values are reported as grand mean ± SEM (standard error of the mean). Pore wall thickness increases with pH, while both pore size and porosity decrease.

| pH | Pore Wall Thickness (µm) (Mean ± SEM) | Pore Size (µm) (Mean ± SEM) | Porosity (%) (Mean ± SEM) |
| --- | --- | --- | --- |
| 6.0 | 0.258 ± 0.044 | 0.439 ± 0.009 | 41.34 ± 2.5 |
| 7.0 | 0.320 ± 0.049 | 0.325 ± 0.016 | 34.66 ± 1.8 |
| 7.4 | 0.633 ± 0.075 | 0.250 ± 0.017 | 32.55 ± 2.3 |
| 8.0 | 0.825 ± 0.037 | 0.214 ± 0.005 | 27.44 ± 2.9 |
| 9.0 | 0.904 ± 0.023 | 0.202 ± 0.020 | 24.55 ± 3.1 |

**Table S2.** The strain-stiffening exponent (*b*) was obtained by fitting differential modulus data (*K*′) versus applied stress (*σ*) to a power-law scaling relationship, *K*′ ∝ *σ^b^*. Log–log plots were generated, and linear regression was applied over the stress range where scaling was observed. The slope of the fit corresponds to *b*, and the regression *R*^2^ indicates the quality of the fit.

| Sample | *b* (stiffening exponent) | *R*^2^ |
| --- | --- | --- |
| PABA_0.20_, pH 9 | 0.20 | 0.97 |
| PABA_0.20_, pH 8 | 0.25 | 0.94 |
| PABA_0.25_, pH 9 | 0.17 | 0.91 |
| PABA_0.25_, pH 8 | 0.26 | 0.99 |
| PABA_0.30_, pH 9 | 0.16 | 0.93 |
| PABA_0.30_, pH 8 | 0.24 | 0.94 |

**Table S3.** Summary of equivalent-circuit (RQ/R) fitting parameters extracted from Bode-plot analysis of Borax–PVA control and PVA-PABA hydrogels, along with the calculated ionic (*σᵢ*) and electronic (*σₑ*) conductivities. *Rᵢ* and *Rₑ* represent the ionic and electronic resistance elements extracted from RQ/R circuit fits to the impedance spectra. *Q* and *n* are constant-phase element parameters describing interfacial dispersion. Ionic and electronic conductivities (*σ*ᵢ and *σ*ₑ) were calculated from the measured sample geometry.

| Sample | *Rᵢ* (Ω) | *Rₑ* (Ω) | *Q* (S·sⁿ) | *n* | *σᵢ* (S m^–1^) | *σₑ* (S m^–1^) |
| --- | --- | --- | --- | --- | --- | --- |
| Borax–PVA, pH 8 | 114.9 ± 16.9 | (2.61 ± 0.65) × 10^8^ | (8.52 ± 0.13) × 10^-7^ | 0.866 ± 0.027 | 5.62 ± 0.82 | (2.56 ± 0.66) × 10^-6^ |
| PABA_0.20_, pH 8 | 129.4 ± 22.2 | (1.51 ± 0.42) × 10^7^ | (8.36 ± 1.54) × 10^-7^ | 0.871 ± 0.041 | 5.02 ± 0.85 | (4.34 ± 1.21) × 10^-5^ |
| PABA_0.25_, pH 8 | 83.1 ± 11.5 | (1.13 ± 0.31) × 10^6^ | (1.38 ± 0.28) × 10^-6^ | 0.793 ± 0.035 | 7.76 ± 1.05 | (5.16 ± 1.31) × 10^-4^ |
| PABA_0.30_, pH 8 | 227.2 ± 28.7 | (2.86 ± 0.55) × 10^5^ | (1.13 ± 0.12) × 10^-5^ | 0.660 ± 0.025 | 2.83 ± 0.36 | (2.28 ± 0.44) × 10^-3^ |

**Table S4.** Equivalent-circuit (RQ/R) fitting parameters and calculated ionic/electronic conductivities of PABA_0.30_ hydrogel before and after 168 h immersion at pH 8. *Rᵢ* and *Rₑ* represent the ionic and electronic resistance elements, respectively, while *Q* and *n* are constant-phase element parameters. The small change in *σᵢ* and *σ*ₑ after immersion indicates that the mixed ionic/electronic transport behavior is largely retained under alkaline aqueous conditions.

| Sample | *Rᵢ* (Ω) | *Rₑ* (Ω) | *Q* (S·sⁿ) | *n* | *σᵢ* (S m^–1^) | *σₑ* (S m^–1^) |
| --- | --- | --- | --- | --- | --- | --- |
| PABA_0.30_, pH 8, before immersion | 375.41 | 5.57 × 10^5^ | 2.13 × 10^-5^ | 0.710 | 1.72 | 1.16 × 10^-3^ |
| PABA_0.30_, pH 8, after 168 h immersion | 455.81 | 6.17× 10^5^ | 4.44 × 10-5 | 0.673 | 1.42 | 1.05 × 10^-3^ |

**Table S5.** Comparison of representative strain-stiffening hydrogels, including network type, charge transport mode, conductivity, and nonlinear stiffening behavior. Most reported systems are electrically insulating or exhibit only ionic conduction, whereas the PVA–PABA hydrogel in this work uniquely combines strain-stiffening with mixed ionic–electronic conductivity.

| System (Ref, Year) | Network Type | Charge Transport Mode | Ionic Conductivity (S m⁻¹) | Electronic Conductivity (S m⁻¹) | Nonlinear Stiffening Metric | Mechanical Range (reported (*G*′), (*G*_0_), or (*E*_0_) | Main Gap / Distinction |
| --- | --- | --- | --- | --- | --- | --- | --- |
| PIC hydrogel (Kouwer et al., 2013)^[4]^ | Semi-flexible fibrillar | Not reported | | | *K*′ ∝ *σ*^3/2^ | (*G*_0_ ~ 10^2^–10^3^) Pa | Strong strain-stiffening but electrically insulating |
| PIC hydrogel (Jaspers et al., 2014)^[5]^ | Semi-flexible fibrillar |  |  |  | (*m* ≈ 1–1.5) | (*G*_0_ ~ 10–10^2^) Pa | Tunable nonlinear mechanics without conductivity |
| PDA fibrous hydrogel (Romera et al., 2017)^[6]^ | Covalent fibrous |  |  |  | (*m* = 1) | (*G*_0_ ~ 4–4000) Pa | Robust fibrous network but insulating |
| PEG–boronate hydrogel (Ollier et al., 2023)^[7]^ | Dynamic-covalent network |  |  |  | (*m* = 0.17–0.49) | (*G*_0_ ~ 10^2^–10^3^) Pa | Dynamic/self-healing behavior without charge transport |
| Hydrazone hydrogel (Beeren et al., 2024)^[8]^ | Dynamic-covalent polymer network |  |  |  | (*m* ≈ 0.7) | (*G*' ~ 10^3^–10^4^) Pa | Chemically tunable but non-conductive |
| PIC-based hybrid hydrogel (Rijns et al., 2024)^[9]^ | Multi-dynamic hybrid network |  |  |  | Tunable stiffening | (*G*' ~ 20–1000) Pa | Multi-component adaptability without conductivity |
| MRH hydrogel (Cui et al., 2022)^[10]^ | Microgel-entangled network | Ionic | ~0.22 | Electrically insulating | J-shaped stiffening | (*E*_0_ = 22–112) kPa | High sensitivity via ionic transport only |
| Patterned hydrogel (Cui et al., 2023)^[11]^ | Hierarchical patterned network | Ionic | ~0.09 | Electrically insulating | ~12–28× modulus increase | (*E*_0_ ~ 31) kPa | Strain-stiffening sensor with protonic conduction |
| PVA–PABA hydrogel (This work) | **Dynamic-covalent polymer network** | **Mixed ionic–electronic** | **~1–10** | **10^-5^–10^-3^** | **(*b* = 0.16–0.26)** | **(*G*_0_ ~ 10–100) Pa** | **Only system combining strain-stiffening with electronic conductivity** |

S4. **References**

[1]	R. E. Daso, R. Posey, H. Garza, A. Perry, C. Petersen, A. C. Fritz, J. Rivnay, J. Tropp, *Adv. Funct. Mater.* **2025**, *35*, 08859.

[2]	J. Schindelin, I. Arganda-Carreras, E. Frise, V. Kaynig, M. Longair, T. Pietzsch, S. Preibisch, C. Rueden, S. Saalfeld, B. Schmid, J.-Y. Tinevez, D. J. White, V. Hartenstein, K. Eliceiri, P. Tomancak, A. Cardona, *Nat. Methods* **2012**, *9*, 676.

[3]	I. Jeon, J. Cui, W. R. K. Illeperuma, J. Aizenberg, J. J. Vlassak, *Adv. Mater.* **2016**, *28*, 4678.

[4]	P. H. J. Kouwer, M. Koepf, V. A. A. Le Sage, M. Jaspers, A. M. Van Buul, Z. H. Eksteen-Akeroyd, T. Woltinge, E. Schwartz, H. J. Kitto, R. Hoogenboom, S. J. Picken, R. J. M. Nolte, E. Mendes, A. E. Rowan, *Nature* **2013**, *493*, 651.

[5]	M. Jaspers, M. Dennison, M. F. J. Mabesoone, F. C. MacKintosh, A. E. Rowan, P. H. J. Kouwer, *Nat. Commun.* **2014**, *5*, 5808.

[6]	M. Fernandez-Castano Romera, R. P. M. Lafleur, C. Guibert, I. K. Voets, C. Storm, R. P. Sijbesma, *Angew. Chem. Int. Ed.* **2017**, *56*, 8771.

[7]	R. C. Ollier, Y. Xiang, A. M. Yacovelli, M. J. Webber, *Chem. Sci.* **2023**, *14*, 4796.

[8]	I. A. O. Beeren, F. L. C. Morgan, T. Rademakers, J. Bauer, P. J. Dijkstra, L. Moroni, M. B. Baker, *J. Am. Chem. Soc.* **2024**, *146*, 24330.

[9]	L. Rijns, M. G. T. A. Rutten, R. Bellan, H. Yuan, M. L. Mugnai, S. Rocha, E. del Gado, P. H. J. Kouwer, P. Y. W. Dankers, *Sci. Adv.* **2024**, *10*, 3209.

[10]	J. Cui, J. Chen, Z. Ni, W. Dong, M. Chen, D. Shi, *ACS Appl. Mater. Interfaces* **2022**, *14*, 47148−47156.

[11]	J. Cui, R. Xu, W. Dong, T. Kaneko, M. Chen, D. Shi, *ACS Appl. Mater. Interfaces* **2023**, *15*, 48736.
